# A multifunctional thermophilic glycoside hydrolase from *Caldicellulosiruptor owensensis* with potential applications in production of biofuels and biochemicals

**DOI:** 10.1186/s13068-016-0509-y

**Published:** 2016-04-30

**Authors:** Xiaowei Peng, Hong Su, Shuofu Mi, Yejun Han

**Affiliations:** National Key Laboratory of Biochemical Engineering, Institute of Process Engineering, Chinese Academy of Sciences, Beijing, China

**Keywords:** β-d-glucosidase, Exoglucanase, β-d-xylosidase, β-d-galactosidase, Transglycosylation, Lignocellulose, Galactooligosaccharides, *Caldicellulosiruptor*

## Abstract

**Background:**

Thermophilic enzymes have attracted much attention for their advantages of high reaction velocity, exceptional thermostability, and decreased risk of contamination. Exploring efficient thermophilic glycoside hydrolases will accelerate the industrialization of biofuels and biochemicals.

**Results:**

A multifunctional glycoside hydrolase (GH) CoGH1A, belonging to GH1 family with high activities of β-d-glucosidase, exoglucanase, β-d-xylosidase, β-d-galactosidase, and transgalactosylation, was cloned and expressed from the extremely thermophilic bacterium *Caldicellulosiruptor owensensis.* The enzyme exerts excellent thermostability by retaining 100 % activity after 12-h incubation at 75 °C. The catalytic coefficients (*k*_cat_/*K*_m_) of the enzyme against *p*NP-β-D-galactopyranoside, *p*NP-β-D-glucopyranoside, *p*NP-β-D-cellobioside, *p*NP-β-D-xylopyranoside, and cellobiose were, respectively, 7450.0, 2467.5, 1085.4, 90.9, and 137.3 mM^−1^ s^−1^. When CoGH1A was supplemented at the dosage of 20 U_cellobiose_ g^−1^ biomass for hydrolysis of the pretreated corn stover, comparing with the control, the glucose and xylose yields were, respectively, increased 37.9 and 42.1 %, indicating that the enzyme contributed not only for glucose but also for xylose release. The efficiencies of lactose decomposition and synthesis of galactooligosaccharides (GalOS) by CoGH1A were investigated at low (40 g L^−1^) and high (500 g L^−1^) initial lactose concentrations. At low lactose concentration, the time for decomposition of 83 % lactose was 10 min, which is much shorter than the reported 2–10 h for reaching such a decomposition rate. At high lactose concentration, after 50-min catalysis, the GalOS concentration reached 221 g L^−1^ with a productivity of 265.2 g L^−1^ h^−1^. This productivity is at least 12-fold higher than those reported in literature.

**Conclusions:**

The multifunctional glycoside hydrolase CoGH1A has high capabilities in saccharification of lignocellulosic biomass, decomposition of lactose, and synthesis of galactooligosaccharides. It is a promising enzyme to be used for bioconversion of carbohydrates in industrial scale. In addition, the results of this study indicate that the extremely thermophilic bacteria are potential resources for screening highly efficient glycoside hydrolases for the production of biofuels and biochemicals.

## Background

Glycoside hydrolases (GHs) are enzymes hydrolyzing the glycosidic bond between carbohydrates or between a carbohydrate and a non-carbohydrate moiety [1]. GHs are widely applied in biological and chemical industries. Among them, cellulases (including endoglucanase, exoglucanase, and β-glucosidase) are of great interest for application in biomass degradation for biofuel and biochemical production [2]. In addition, some GHs have the capability of transglycosylation and are used for synthesis of oligosaccharides and glycosides, such as galactooligosaccharides (GalOS) (synthesized by β-galactosidase) [3] and octyl glucoside (synthesized by β-glucosidase) [4]. Production of efficient GHs is urgently required to improve the economical possibility for the related industrial products. Especially, efficient cellulase production has developed into a significant bottleneck for the biofuel industry [1]. Research efforts have recently focused on extremely thermophilic microorganisms for exploring novel cellulases and other GHs to improve the current situation [5–7], due to the advantages of these thermophilic enzymes, such as higher reaction velocity, excellent thermostability, and decreased risk of contamination [8]. Many thermophilic GHs, such as β-d-glucosidase [9, 10], bifunctional cellulolytic enzyme (endo- and exoglucanases) [11], β-d-xylosidase [12], and β-d-galactosidase [3, 13], have been cloned, heterologously expressed, and biochemically characterized for the purpose of uncovering the catalytic mechanism and evaluating the possibility of industrial applications. Among them, the genus *Caldicellulosiruptor* has recently attracted high interest for it can produce a diverse set of glycoside hydrolases (GHs) for deconstruction of lignocellulosic biomass [7, 14, 15]. The open *Caldicellulosiruptor* pangenome encoded 106 glycoside hydrolases (GHs) from 43 GH families [7]. Our previous work [16] found that the extremely thermophilic bacterium *Caldicellulosiruptor owensensis* had comprehensive hemicellulase and cellulase system with potential application for bioconversion of lignocellulosic biomass. Moreover, the catalytic mechanism of some enzymes from *Caldicellulosiruptor* differs from the general GHs. For example, the cellulase CelA produced from *Caldicellulosiruptor bescii* could hydrolyze the microcrystalline cellulose not only from the surface as common cellulases done but also by excavating extensive cavities into the surface of the substrate [17]. The information about their genetic, biochemical, and biophysical characteristics suggests that there exist more efficient GHs to be explored in the extremely thermophilic microorganisms *Caldicellulosiruptor*.

In the present work, a multifunctional glycoside hydrolase (GH) was cloned and expressed from *C. owensensis.* The characteristics of the enzyme and its potential applications in saccharification of lignocellulosic biomass and synthesis of galactooligosaccharides (GalOS) were evaluated.

## Results and discussion

### Cloning and expression of CoGH1A

**Fig. 1:**
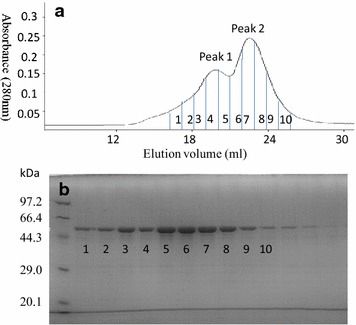
Gel filtration chromatography and SDS-PAGE analysis of CoGH1A. **a** Quaternary structure analysis of CoGH1A by gel filtration chromatography. **b** SDS-PAGE of CoGH1A fractions collected from gel filtration chromatography. The bands marked with *Arabic numerals 1–10* corresponded to eluents from **a**

The gene Calow_0296 consists of a 1359 bp fragment encoding 452 amino acids, which belongs to glycoside hydrolase family 1 (GH1) and was named CoGH1A. The predicted molecular weight of CoGH1A was 53.2 kDa. The SDS-PAGE analysis agreed with the predicted sizes (Fig. 1b). The quaternary structure of purified CoGH1A was analyzed through gel filtration chromatography coupled with SDS-PAGE. The results are shown in Fig. 1. The molecular mass (MW) of CoGH1A at peak 1 (160.4 kDa) was almost three times that at peak 2 (53.3 kDa) (Fig. 1a). However, fractions collected from two peaks showed the same band in SDS-PAGE (Fig. 1b). This suggests that CoGH1A exists as monomer and homotrimer in buffer. Two thermostable β-glucosidases, one belonging to GH1 from the Termite *Nasutitermes takasagoensis* [18] and the other belonging to GH3 from *Thermoascus aurantiacus* [19], were also homotrimers but not monomers in their native form. It is interesting that both monomer and homotrimer of CoGH1A exist in native form. It seems that monomer and homotrimer collectively function on the substrate.

### Optimum temperature, pH and thermostability of CoGH1A

**Fig. 2:**
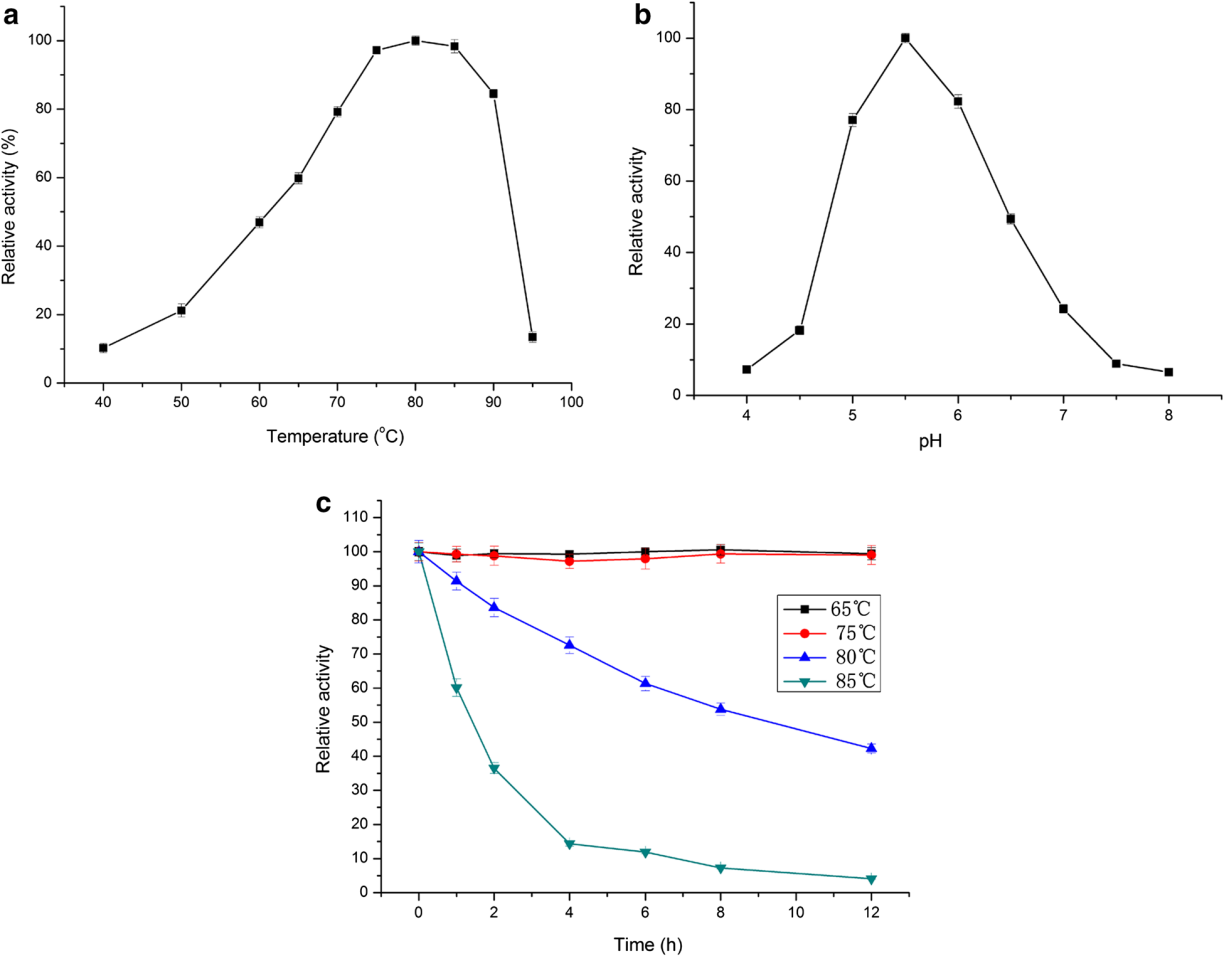
Effect of temperature (**a**) and pH (**b**) on activity and thermostability (**c**) of CoGH1A. Values are averages counted from three independent measures; *error bars* represent standard deviation

The effects of temperature and pH on the activity of CoGH1A using *p*NP-β-d-galactopyranoside as the substrate are shown in Fig. 2a, b. It shows that the optimum temperature of CoGH1A was from 75 to 85 °C, which is in accordance with the optimum temperature for *C. owensensis* growth at 75 °C [20]. At 70 and 90 °C, the activities of CoGH1A were more than 80 % of the maximum, while below 60 °C the enzyme activity decreased to less than 50 % of the maximum. This indicates that CoGH1A is an extremely thermophilic enzyme and has broad temperature adaptability. The optimum pH of CoGH1A was 5.5. At the pH of 5.0 and 6.0, the activities of CoGH1A were about 80 % of the maximum. At the pH of 4.5 and 7.0, the activities of CoGH1A were deceased to about 20 % of the maximum. It is better to control the pH from 5.0 to 6.0 for application of CoGH1A. This is a common pH for most of the glycoside hydrolases.

The thermostability of CoGH1A is shown in Fig. 2c. After 12 h of incubation in pH 5.5 citrate buffer at 65 and 75 °C, the activities of the enzyme remained the same as that of the initial. These results show that CoGH1A exhibits excellent thermostability at the temperatures below 75 °C. The half-lives of CoGH1A were about 11 and 1.5 h when cultivated at 80 and 85 °C, respectively. The half-lives of the β-glucosidase CbBgl1A from *C. bescii* [9] at 80 and 85 °C were 20 and 8 min, respectively. The half-lives of the β-galactosidase from *C. saccharolyticus* [21] at 75 and 80 °C were 17 and 2 h, respectively. This indicates that among the enzymes from *Caldicellulosiruptor* species CoGH1A is a robust candidate for industrial application.

### Effect of ions on the activity of CoGH1A

The effect of cations on the activity of CoGH1A was detected at pH 5.5 and 75 °C (Table 1). On the whole, at the concentrations of both 5 and 10 mM, the effects of cations on the activity of CoGH1A were not significant. Mn^2+^ and Ni^2+^ activated the enzyme activity by 135 and 119 %, respectively, at the concentration of 10 mM. K^+^ activated the enzyme activity by 114 and 105 %, respectively, at the concentrations of 5 and 10 mM. The other cations, such as Fe^3+^, Zn^2+^, Co^2+^, Mg^2+^, Cu^2+^, Na^+^, and NH_4_^+^, slightly inhibited the enzyme activity by 71–96 %. These results indicate that CoGH1A has resistance to cations and additional cation is not necessary for activating the enzyme. The effects of cation on CoGH1A were similar to those on some other glycoside hydrolases from *Caldicellulosiruptor* species, such as the xylanase from *C. kronotskyensis* [22] and the xylanase and xylosidase from *C. owensensis* [12]. They were very different from the β-galactosidase produced by *Lactobacillus delbrueckii* [23]. The K^+^ and Na^+^ activated that β-galactosidase with the activities increased almost 5- and 12-fold, respectively, at the ion concentration of 50 mM, while Zn^2+^ significantly inhibited the activity of that β-galactosidase with the activity decreased to almost zero at the Zn^2+^ concentration of 10 mM.

**Table 1:** Effect of cations on the activity of CoGH1A

Cations	Relative activity %	
	5 mM	10 mM
Control	100	100
Fe^3+^	92	83
Zn^2+^	87	82
Ni^2+^	102	119
Mn^2+^	104	135
Co^2+^	95	75
Mg^2+^	76	80
Cu^2+^	87	71
K^+^	114	105
Na^+^	96	82
NH_4_^+^	82	75

The experiment was carried out at 75 °C and pH 5.5. The *p*NPGal with the concentration of 1 mM was used as the substrate

### Specific activities and kinetic parameters of CoGH1A on different substrates

The activities of CoGH1A on various substrates were tested at 75 °C and pH 5.5. The results (Table 2) show that the enzyme exhibited broad substrate specificity. The highest specific activity was 3215 U mg^−1^ with *p*NP-β-d-galactopyranoside (*p*NPGal) as the substrate, followed by 1621, 603, 280, 140, and 130 U mg^−1^ with *p*NP-β-d-glucopyranoside (*p*NPGlu), *p*NP-β-d-cellobioside (*p*NPC), lactose, *p*NP-β-d-xylopyranoside (*p*NPX), and cellobiose, respectively, as substrates. Also, the enzyme displayed activities on soluble polysaccharides such as synanthrin (2.4 U mg^−1^) and locust bean gum (1.2 U mg^−1^). The enzyme even hydrolyzed insoluble substrates with slight activities on cotton (<0.1 U mg^−1^) and filter paper (<0.1 U mg^−1^). However, it exhibited no activity against carboxymethyl cellulose (CMC) and *p*NP- α-l-arabinofuranoside (*p*NPAr).

**Table 2:** Specific activities of CoGH1A on different substrates

Substrate	Specific activity (U mg^−1^)
*p*NPGal^a^	3215 ± 6.2
*p*NPGlu^a^	1621 ± 15.6
*p*NPC^a^	603 ± 11.4
*p*NPX^a^	140 ± 5.4
Cellobiose^a^	130 ± 3.6
Lactose^a^	280 ± 4.2
Locust bean gum^b^	2.4 ± 0.2
Synanthrin^b^	1.2 ± 0.1
Cotton^b^	<0.1
Filter paper^b^	<0.1
*p*NPAr^a^	ND
CMC^b^	ND

*ND* not detected

^a^Initial concentration was 1 mM

^b^Initial concentration was 1 % (W/V)

The kinetic parameters of CoGH1A were determined on several preferred substrates at 75 °C and pH 5.5 (Table 3). The *K*_m_ values against *p*NPGal, *p*NPGlu, *p*NPC, pNPX, and cellobiose were 0.61, 1.52, 0.87, 7.18, and 15.65 mM, respectively. The catalytic coefficients (*k*_cat_/*K*_m_) on the five substrates were, respectively, 7450.0, 2467.5, 1085.4, 90.9, and 137.3 mM^−1^ s^−1^.

**Table 3:** Kinetic parameters of CoGH1A on different substrates

Substrate	*K*_m_ (mM)	*V*_max_ (μmol mg^−1^ min^−1^)	*k*_cat_ (s^−1^)	*k*_cat_/*K*_m_ (mM^−1^ s^−1^)
*p*NPGal	0.61 ± 0.025	5100 ± 64	4522 ± 56.7	7450.0
*p*NPGlu	1.52 ± 0.2	4027 ± 75	3750.6 ± 66.5	2467.5
*p*NPC	0.87 ± 0.12	1065 ± 38	944.3 ± 33.7	1085.4
*p*NPX	7.18 ± 1.4	736 ± 35	652.6 ± 31.0	90.9
Cellobiose	15.65 ± 1.52	2424 ± 48	2149 ± 43	137.3

A complete cellulase system consists of at least three related enzymes: endoglucanases (EC 3.2.1.4), exoglucanases (or cellobiohydrolase) (EC 3.2.1.91), and β-glucosidases (EC 3.2.2.21) [24]. They cooperate in releasing glucose. Endoglucanases (EC 3.2.1.4) randomly hydrolyze internal glycosidic bonds to decrease the length of the cellulose chain and multiply polymer ends. Exoglucanases (EC 3.2.1.91) split-off cellobiose or glucose from cellulose termini and β-glucosidases (EC 3.2.2.21) hydrolyze cellobiose and oligomers to render glucose [24]. Among them, β-glucosidases are essential for efficient hydrolysis of cellulosic biomass as they relieve the inhibition of the cellobiohydrolases and endoglucanases by reducing cellobiose accumulation [25]. Commercial cellulase preparations are mainly based on mutant strains of *Trichoderma reesei* which have usually been characterized by a low secretion of β-glucosidase [26]. Thus, *T. reesei* cellulase preparations had to be supplemented with added β-glucosidase to provide the more efficient saccharification of cellulosic substrates [26, 27]. Much has been devoted to finding highly efficient β-glucosidases for bioconversion of cellulosic substrates. Some of the β-glucosidases with relatively high activity are listed in Table 4. The *V*_max_ of CoGH1A was 4027 ± 75 and 2424 ± 48 μmol mg^−1^ min^−1^ with *p*NPGlu and cellobiose, respectively, as substrates, which was lower than that of the β-glucosidase from *Pholiota adipose* with the *V*_max_ of 4390 and 3460 μmol mg^−1^ min^−1^ using *p*NPGlu and cellobiose, respectively, as substrates [28]. However, the thermostability of CoGH1A was better than that of the β-glucosidase from *Pholiota adipose*. The former kept 100 % activity after incubated at 75 °C for 12 h (Fig. 2c) and the latter kept 50 % activity after incubated at 70 °C for 8.5 h [28]. The catalytic coefficient (*k*_cat_/*K*_m_) of the β-glucosidase from the hyperthermophilic bacterium *Thermotoga petroph* was 30,800 mM^−1^ s^−1^ with *p*NPGlu as a substrate [10]. To the best of our knowledge, this is by far the highest reported β-glucosidase activity with *p*NPGlu as a substrate. However, the capability of this β-glucosidase for hydrolysis of cellobiose was very low, with the specific activity of only 2.3 U mg^−1^ [10]. This will limit its application in saccharification of lignocellulosic biomass. Another β-glucosidase from the same genus bacterium strain *C. bescii* was studied in detail [9]. The catalytic coefficients (*k*_cat_/*K*_m_) of that β-glucosidase were 84.0 and 87.3 mM^−1^ s^−1^ with *p*NPGlu and cellobiose, respectively, as the substrate [9], which were much lower than those (2467.5 and 137.3 mM^−1^ s^−1^) of CoGH1A. These results show that CoGH1A is a potential β-glucosidase candidate for industrial application.

**Table 4:** CoGH1A and some reported microbial β-glucosidases with relatively high activity

Resource	GH family	Substrate	*K*_m_ (mM)	*V*_max_ (μmol mg^−1^ min^−1^)	*k*_cat_ (s^−1^)	*k*_cat_/*K*_m_ (mM^−1^ s^−1^)	OT(°C)	OP	References
*C. owensensis*	GH1	*p*NPGlu	1.52	4027	3750.6	2467.5	75–85	5.5	This work
		Cellobiose	15.65	2424	2149	137.3			
*C. bescii*	GH1	*p*NPGlu	3.71	–	311.6	84.0	85	6.8	[9]
		Cellobiose	90.8	–	757.9	8.35			
*Neosartorya fischeri*	GH1	*p*NPGlu	68	886	826.7	12.2	40	6	[46]
*Pholiota adipose*	GH3	*p*NPGlu	2.23	4390	–	–	65	5	[28]
		Cellobiose	5.60	3460	–	–			
*Fomitopsis pinicola*	GH3	*p*NPGlu	1.8	1710	2990	1700	50	4.5	[47]
*Thermotoga petroph*	GH1	*p*NPGlu	2.8	42,700	87,400	30,800	80–90	7–8	[10]
*Stereum hirsutum*	GH1	*p*NPGlu	2.5	3028	–	–	65	4.5	[48]
		Cellobiose	86	172	–	–			
*Talaromyces thermophilus*	–	*p*NPGlu	0.25	228.7	–	–	65	5.0	[49]
		Cellobiose	16.7	1519.1	–	–			

*OT* optimum temperature; *OP* optimum *pH*; *–* no data

Exoglucanases or cellobiohydrolases (CBHs) preferentially hydrolyze β-1, 4-glycosidic bonds from chain ends, producing cellobiose as the main product. CBHs have been shown to create a substrate-binding tunnel with their extended loops which surround the cellulose [29, 30]. Besides microcrystalline cellulose and cotton cellulose, the *p*NPC was also used as a substrate for detecting the exoglucanases or cellobiohydrolase (CBH) activity [31, 32]. The specific activity and *V*_*max*_ of CoGH1A were, respectively, 603 U mg^−1^ and 1065 μmol mg^−1^ min^−1^ using *p*NPC as the substrate (Tables 2, 3), which were much higher than those for most of the reported enzymes. For example, Le et al. purified a novel cellobiohydrolase from *Penicillium decumbens* with a specific activity of 1.9 U mg^−1^ against *p*NPC [32]. Lee et al. purified a cellobiohydrolase from *Penicillium purpurogenum* with a specific activity of 10.8 U mg^−1^ against *p*NPC U mg^−1^ [33]. Bok et al. [11] purified two enzymes (CelA and CelB) with both endo- and exoglucanase activities. The *V*_max_ of CelA and CelB were, respectively, 69.2 and 18.4 μmol mg^−1^ min^−1^ with *p*NPC as a substrate. The high specific activity against *p*NPC indicates that the CoGH1A has high ability for split-off cellobiose or split-off glucose one by one from *p*NPC. However, using filter paper or cotton as the substrate the activities were very low (<0.1 U mg^−1^, Table 2). This is not surprising because the CoGH1A has only catalytic domain (CD) without carbohydrate-binding module (CBM) which would facilitate the enzyme binding to the substrate. Therefore, CoGH1A works primarily on soluble oligosaccharides.

β-galactosidases catalyze the hydrolysis of the β-1, 4-D glycosidic linkage of lactose and structurally related substrates. β-galactosidases have two main technological applications in the food industry, namely the removal of lactose from milk and dairy products [34] and the production of galactooligosaccharides (GalOS) from lactose by transglycosylation [3].

CoGH1A exhibits very high β-galactosidase activity with the catalytic coefficient (*k*_cat_/*K*_m_) of 7450.0 mM^−1^ s^−1^ on *p*NPGal. This is by far the highest catalytic coefficient for all reported β-galactosidases on *p*NPGal (Table 5), much higher than the second high catalytic coefficient of 1462.8 mM^−1^ s^−1^ by the enzyme from the thermoacidophilic bacterium *Alicyclobacillus acidocaldarius* [35]. The catalytic coefficient of another β-galactosidase from the same genus strain *Caldicellulosiruptorsaccharolyticus* was 149 mM^−1^ s^−1^ [21], much lower than that of CoGH1A, although the optimum temperature and pH between them are similar. Table 5 also shows that most of the β-galactosidases with higher activity belong to GH1. The optimum pH and temperature of these GH1 β-galactosidases are, respectively, 5.5–6.5 and 65–85 °C. In general, the higher temperatures can be beneficial in higher oligosaccharide yields. The problem of microbial contamination can also be solved by increasing the catalysis temperature [34]. CoGH1A with high β-galactosidase activity and high optimum temperature (75–85 °C) will be a promising enzyme in the food industry.

**Table 5:** Kinetic parameters of CoGH1A and some reported microbial β-galactosidases with relatively high activity

Resource	GH family	Substrate	*K*_m_ (mM)	*V*_max_ (μmol mg^−1^ min^−1^)	*k*_cat_ (s^−1^)	*k*_cat_/*K*_m_ (mM^−1^ s^−1^)	OT (°C)	OP	Reference
*C. owensensis*	GH1	*p*NPGal	0.607	5100	4522	7450	75–85	5.5	This work
*Alicyclobacillus acidocaldarius*	GH1	*o*NPGal	5.50	–	8045.5	1462.8	65	5.5	[35]
*Hot spring metagenome*	GH1	*p*NPGal	3.33	40.00	2000.0	600.6	65	8.0	[50]
*Lactobacillus delbrueckii* subsp. *bulgaricus*	GH2	*o*NPGal	0.919	317	603	655	45–60	7.5	[23]
		Lactose	19.2	123	234	12.3			
*Bifidobacterium breve*	GH2	*o*NPGal	1.3	486	937	722	50	7.0	[51]
Marine metagenomic library	GH2	Lactose	17.98	–	131.16	7.29	50	7.0	[52]
*Bacillus licheniformis*	GH42	*o*NPGal	13.7	299	785	57.3	50	6.5	[53]
*C. saccharolyticus*	GH42	*o*NPGal	1.21	–	149	123	80	6.0	[21]
		Lactose	30.0	–	42	1.4			
*Thermotoga naphthophila*	GH42	*o*NPGal	1.31	3385.67	–	–	90	6.8	[13]
		Lactose	1.43	2.67	–	–	70	5.8	
*Thermotoga maritima*	GH42	*p*NPGal	2.74	351	5.85	2.13	80	5.5	[54]

*OT* optimum temperature; *OP* optimum *pH*; *–* no data

### Supplementation of CoGH1A for lignocellulosic biomass hydrolysis

**Fig. 3:**
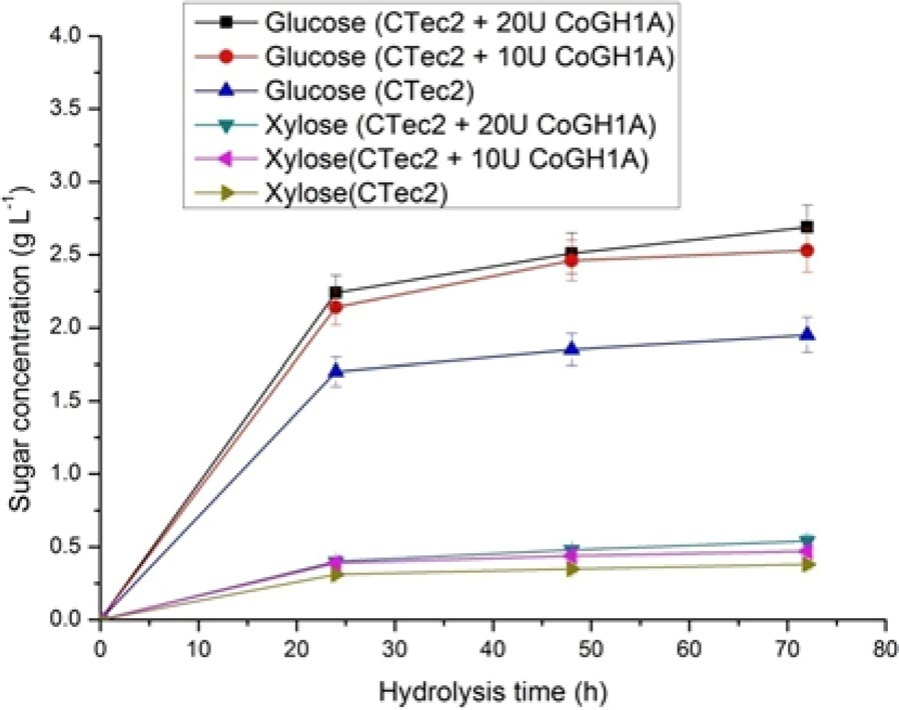
Synergetic hydrolysis of steam-exploded corn stover by CTec2 and CoGH1A. This experiment was carried out in pH 5.5 acetate buffer at 60 °C with the initial biomass of 20 g L^−1^. The CTec2 loading rate was 10 FPU g^−1^ biomass. The loading rate of the supplemented CoGH1A was 10 or 20 U_cellobiose_ g^−1^ biomass. Values are averages counted from three independent measures; *error bars* represent standard deviation

Based on the fact that CoGH1A is a multifunctional glycoside hydrolase, especially with high β-glucosidase activity, its capacity on saccharification of lignocellulosic biomass was determined. The experiment for hydrolysis of the steam-exploded (SE) corn stover by the commercial enzyme CTec2 (Novozymes) supplemented with CoGH1A was carried out at 60 °C and pH 5.5. As shown in Fig. 3, supplementation of CoGH1A could markedly enhance saccharification of the steam-exploded (SE) corn stover. Supplementing CoGH1A with the dose of 10 and 20 U_cellobiose_ g^−1^ biomass, after 72-h hydrolysis, the concentrations of glucose increased from 1.95 (hydrolyzed by CTec2 only) to 2.53 and 2.69 g L^−1^, increasing 29.7 and 37.9 %, respectively. In addition, the concentrations of xylose also increased from 0.38 to 0.47 and 0.54 g L^−1^, increasing 23.7 and 42.1 %, respectively. This indicates that CoGH1A contributed not only for cellulose but also for hemicellulose hydrolysis, due to its multi-activities on glycoside hydrolysis (Table 2 and 3). Although the catalytic temperature 60 °C in this experiment was not optimum for both CTec2 (50 °C) and CoGH1A (75–85 °C), especially, the activity of CoGH1A at 60 °C was only about 50 % of the maximum (Fig. 2), this experiment demonstrated the high ability of CoGH1A on saccharification of lignocellulosic biomass. CoGH1A, possessing β-glucosidase, exoglucanase, and β-xylosidase activities, may be a promising enzyme for industrial application in bioconversion of lignocellulosic biomass.

### Lactose transformation and synthesis of galactooligosaccharides

**Fig. 4:**
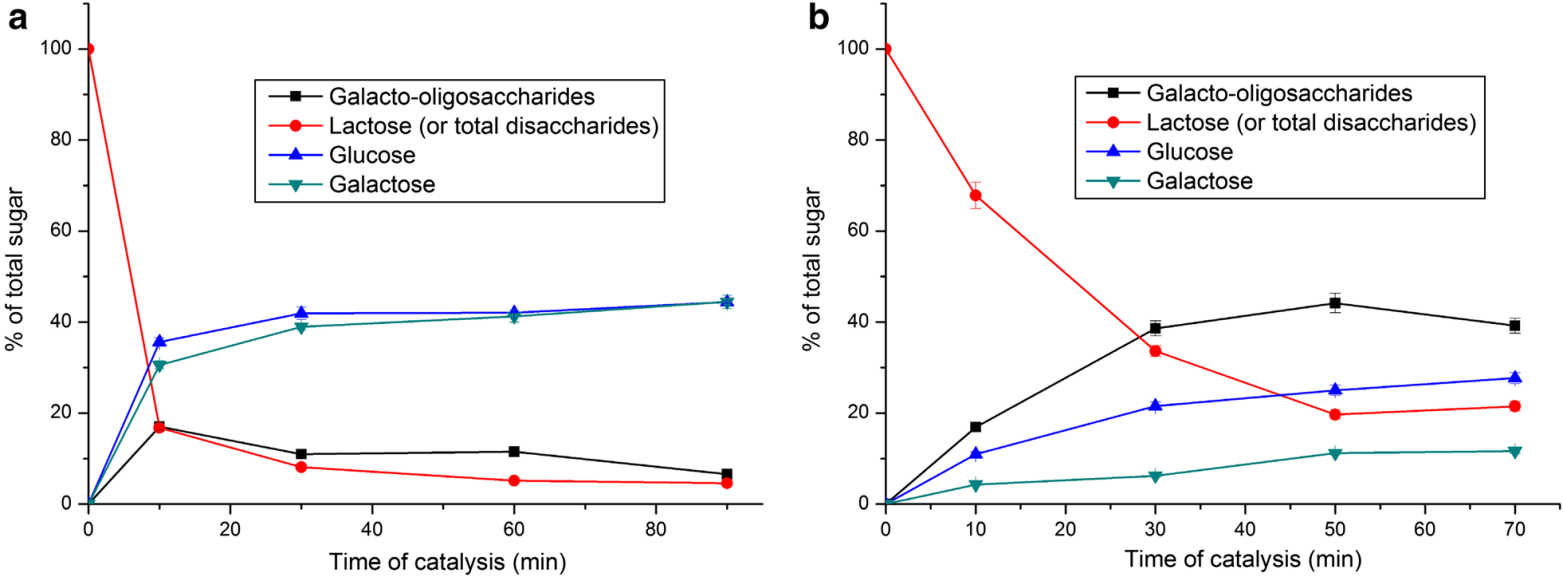
Time course of the enzymatic catalysis of lactose degradation and GalOS synthesis by CoGH1A at the initial lactose concentration of 40 g L^−1^ (**a**) and 500 g L^−1^ (**b**). Values are averages counted from three independent measures; *error bars* represent standard deviation

Since CoGH1A has very high β-galactosidase activity, its potential industrial applications were further investigated in low (40 g L^−1^) and high (500 g L^−1^) initial lactose concentrations by cultivating in pH 5.5 citrate buffer at 70 °C with the enzyme dosage of 2.5 U_lactose_ mL^−1^. Figure 4a shows the time course of the enzymatic catalysis at the initial lactose concentration of 40 g L^−1^ which is very close to the concentration of lactose in fresh milk. After 10-min reaction, more than 83 % of the lactose was degraded and the yields of GalOS, glucose, and galactose were 17.0, 35.6, and 30.6 % with the concentrations of 6.80, 14.24, and 12.24 g L^−1^, respectively. When the reaction continued to 30 min, about 92 % of the lactose was converted and the yields of GalOS, glucose, and galactose were 11.0, 41.9, and 39.0 % with the concentrations of 4.40, 16.46, and 15.6 g L^−1^, respectively. Further prolonging the reaction time, the concentrations of lactose and GalOS were gradually decreased, while the concentrations of glucose and galactose were gradually increased.

In literature, when the lactose hydrolysis rates reached 70–95 %, the reaction time needed is 2–10 h [34, 36–38]. However, in this research only 10 min was needed by CoGH1A. The high efficiency of CoGH1A makes it a potential candidate in milk and milk product industries. Moreover, CoGH1A has a function in the synthesis of GalOS, with 10–20 % GalOS yield during lactose hydrolysis. This will upgrade the quality and value of milk and milk products since GalOS are non-digestible oligosaccharides and are used as prebiotic food ingredients.

When the initial lactose concentration was 500 g L^−1^ (Fig. 4b), the GalOS yield was much higher than that with low initial lactose concentration (40 g L^−1^ Fig. 4a). This is in agreement with the fact that higher initial lactose concentrations can be beneficial in higher oligosaccharide yields [34]. The GalOS yield was 38.6 % after 30-min catalysis and gradually increased to 44.2 % at 50 min. Further catalyzing for 70 min, the GalOS yield was slightly decreased to 39.2 %. This indicated that reaction for 50 min is enough for achieving the highest GalOS yield. The highest GalOS concentration reached 221 g L^−1^ with a productivity of 265.2 g L^−1^ h^−1^.

Figure 4 also shows that at low initial lactose concentration (Fig. 4a) the glucose and galactose were the main products (totally about 80 %) and the yield of glucose was almost the same as that of galactose. This indicates that the velocity of lactose decomposition was much higher than that of GalOS synthesis in the condition of low initial lactose concentration. During the catalytic process at high initial lactose concentration (Fig. 4b), the concentration of lactose was quickly decreased. It was decreased more than 80 % after 50 min; however, the yield of galactose, which was produced from lactose decomposition, was kept below 11.6 % because galactose was used for GalOS synthesis. It shows that the velocity of lactose decomposition was almost same as that of GalOS synthesis in the condition of high initial lactose concentration.

The characteristics of GalOS synthesis by CoGH1A at the initial lactose concentration of 500 g L^−1^ and some other β-galactosidases in batch process are shown in Table 6. The GalOS productivity by CoGH1A was more than 12 times higher than that of the β-galactosidases from *Lactobacillus delbrueckii* subsp. *Bulgaricus* (19.8 g L^−1^ h^−1^) [23], *Bifidobacterium breve* (14.7 g L^−1^ h^−1^) [51], marine metagenomic library (20.6 g L^−1^ h^−1^) [52], and *Thermotoga maritima* (18.20 g L^−1^ h^−1^) [39]. Time for reaching the maximum yield by the four β-galactosidases was 5–10 h (Table 6), which is at least fivefold longer than that by CoGH1A. Although the enzyme loading rate of CoGH1A (2.5 U_lactose_ mL^−1^) in this study was higher than that from *Lactobacillus delbrueckii* subsp. *bulgaricus* (1.5 U_lactose_ mL^−1^) and *Thermotoga maritima* (1.5 U_lactose_ mL^−1^), the actual protein loading rate of CoGH1A was 8.9 μg mL^−1^ which was much lower than those of other β-galactosidases (Table 6). Moreover, CoGH1A has high thermostability with retaining 100 % activity after incubation at 75 °C for 12 h (Fig. 2c). The problem of microbial contamination can also be alleviated or avoided at such a high temperature. These advantages of CoGH1A make it a potential candidate for GalOS synthesis.

**Table 6:** Characteristic of GalOS synthesis by different β-galactosidases in batch process

Enzyme source	Initial lactose concentration (g L^−1^)	Enzyme loading (U_lactose_ ml^−1^ μg ml^−1^)		Temperature (°C)	Time for reaching maximum yield	GalOS productivity (g L^−1^ h^−1^)	Maximum yield of GalOS %	References
*Lactobacillus delbrueckii* subsp. *bulgaricus*	205	1.5	>12.2^a^	50	5 h	19.8	~50	[23]
*Bifidobacterium breve*	200	2.5	>25.8^a^	30	6 h	14.7	44	[51]
*Thermotoga maritima*	500	1.5	>23.6^a^	80	5 h	18.2	18	[39, 55]
Marine metagenomic library	360	18	267.9	40	~10 h	20.6	57.1	[52]
*Caldicellulosiruptor owensensis*	500	2.5	8.9	70	50 min	265.2	44.2	This work

^a^The given data were calculated using the values of the *V*_max_ of each enzyme as the specific activity

### Multiple sequence alignment and the possible role in host bacterium

**Fig. 5:**
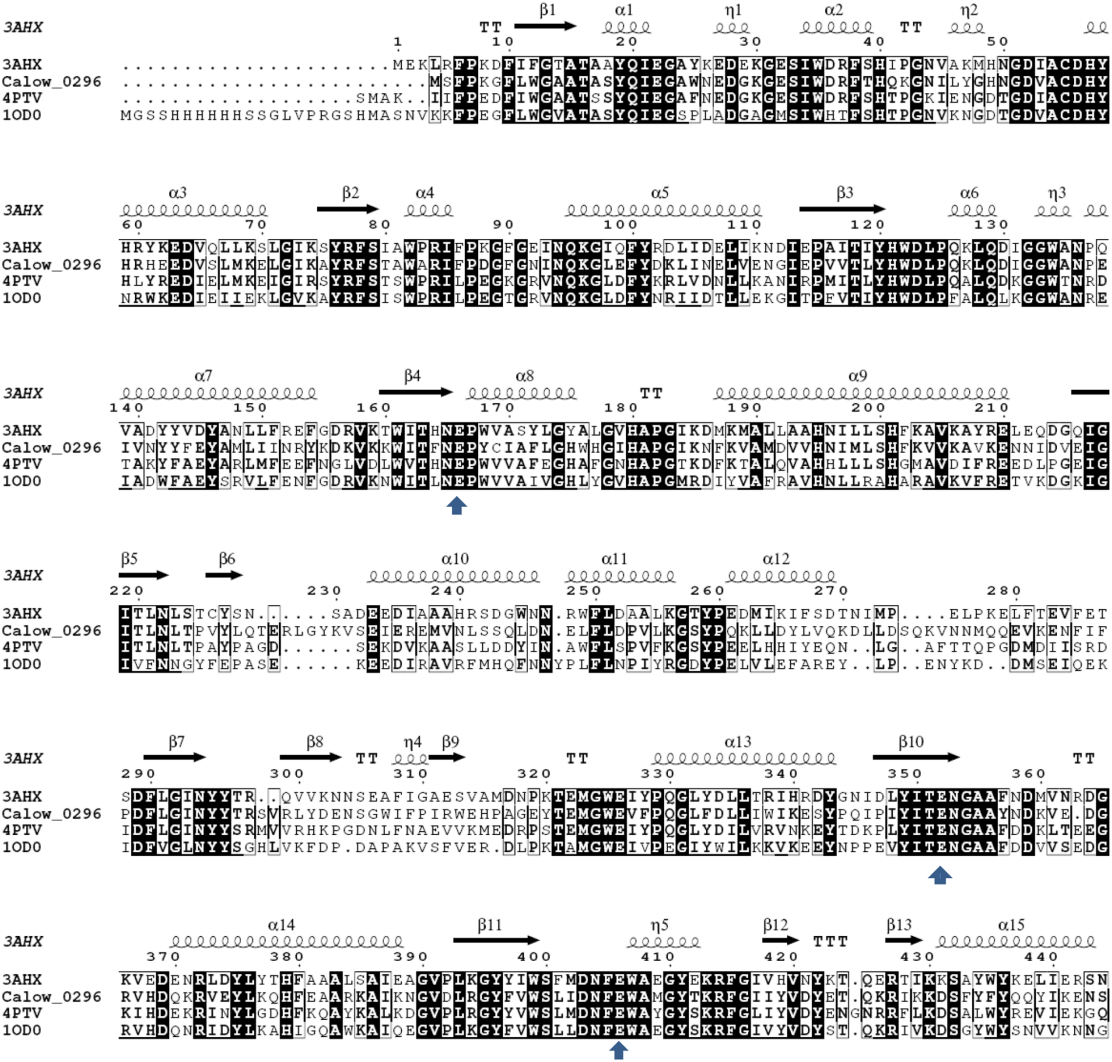
Multiple sequence alignment of Calow_296 and the three resolved proteins with relatively higher identity. The *blue arrows* indicate the conserved catalytic glutamic acid residues

The structure and active site residues of the enzyme were predicted for analyzing the difference between CoGH1A and other GHs whose structures were resolved. Three proteins with relatively higher identity to CoGH1A were found, i.e., 3AHX (a β-glucosidase from *Clostridium cellulovorans*) [40], 4PTV (a β-glucosidase from *Halothermothrix orenii*) [41], and 1OD0 (a β-glucosidase from *Thermotoga maritima*) [42]. CoGH1A shares, respectively, 53, 50, and 48 % identity with them. However, the catalytic coefficients (*k*_cat_/*K*_m_) of 3AHX, 4PTV, and 1OD0 were, respectively, 340, 187, and 102 mM^−1^ s^−1^ with *p*NPGlu (for 3AHX and 4PTV) [40, 41] or 2, 4-dinitrophenyl-β-D-glucopyranoside (for 1OD0) [42] as a substrate. They are much lower than those of CoGH1A. Multiple sequence alignment of CoGH1A with the aforesaid proteins (Fig. 5) was performed using the ClustalX2 software and depicted online by ESPrit 3.0 (http://www.espript.ibcp.fr/). The catalytic acid/base and catalytic nucleophile of CoGH1A are predicted as Glu163 and Glu361 (pointed out by blue arrows in Fig. 5), respectively. The residual Glu414 is predicted to be participated in substrate binding. As Fig. 5 shows, both the catalytic glutamic acid residues and the amino acid residues next to them are conserved among the four enzymes. This suggests that the catalytic coefficients of these enzymes are determined by the amino acid sequences except the conserved catalytic glutamic acids.

*Caldicellulosiruptor owensensis*, the host bacterium of CoGH1A, was isolated from a shallow freshwater pond located in the Owens Lake bed area [20]. It could grow on a wide variety of carbon sources including arabinose, glucose, cellobiose, cellulose, xylan, xylose, galactose, dextrin, fructose, lactose, glycogen, inositol, mannitol, mannose, maltose, pectin, raffinose, rhamnose, ribose, starch, sucrose, and tagatose [20]. In its growing environment, the polysaccharides (or glycans) may be the most possible carbon sources that *C. owensensis* could be obtained. Decomposition of these polysaccharides to monoses is necessary for *C. owensensis* to assimilate them. The multifunctional glycoside hydrolase CoGH1A, which could efficiently convert various soluble oligosaccharides to monoses, may give important contribution to *C. owensensis* for its capability of utilizing the wide variety of carbon sources. The thermostability of CoGH1A and its resistance to cations may also help the host bacterium growing in the changeful environments.

## Conclusions

A multifunctional glycoside hydrolase, named CoGH1A, has been cloned and expressed from the extremely thermophilic bacterium *C. owensensis.* It possesses high activities of β-d-glucosidase, exoglucanase, β-d-xylosidase, β-d-galactosidase, and transgalactosylation. Moreover, it exerts excellent thermostability by retaining 100 % activity after 12-h incubation at 75 °C. The enzyme contributed not only for glucose but also for xylose release when it was supplemented for hydrolysis of corn stover. Additionally, the catalytic efficiencies of this enzyme on lactose decomposition and galactooligosaccharides synthesis were at least, respectively, 5- and 12-fold those of the reported glycoside hydrolases. The multifunctional glycoside hydrolase CoGH1A is a promising enzyme to be used for bioconversion of biomass and carbohydrate. This research also indicates that the extremely thermophilic bacteria are potential resources for screening highly efficient GHs for the production of biofuels and biochemicals.

## Methods

### Materials

The extremely thermophilic cellulolytic bacteria *C. owensensis* DSM 13,100 was purchased from the DSMZ (German Collection of Microorganisms and Cell Cultures). The substrates, *p*-nitrophenyl β-d-galactopyranoside (*p*NPGal), *p*-nitrophenyl β-d-glucopyranoside (*p*NPGlu), *p*-nitrophenyl β-d-cellobioside (*p*NPC), *p*-nitrophenyl β-d-xylopyranoside (*p*NPX), *p*-nitrophenyl β-d-mannopyranoside (*p*NPM), *p*-nitrophenyl α-l-arabinofuranoside (*p*NPAr), carboxymethylcellulose (CMC), locust bean gum, and synanthrin, were purchased from Sigma. The chemicals and other substrates were purchased from Sinopharm Chemical Reagent Beijing Co., Ltd or Sigma. The steam-exploded corn straw was obtained by being pretreated in the condition of 1.5 MPa retained for 3 min. Its composition was glucan 46.8 %, xylan 4.3 %, araban 0 %, and lignin 27.4 %. Competent cells used for cloning and expression were *Escherichia coli* Top10 (TianGen, China) and *E. coli* BL21 (DE3), respectively. The *E.coli* BL21 (DE3) competent cells were prepared with the methods described in Molecular Cloning: A Laboratory Manual [43].

### Cloning the gene of CoGH1A

*Caldicellulosiruptor owensensis* cells were inoculated at 75 °C, 75 rpm for 24 h with the DSMZ medium 640 (detailed list of ingredient was supplied by DSMZ, http://www.dsmz.de). The genomic DNA of *C. owensensis* was extracted from 3 mL of fermentation broth using TIANamp Bacteria DNA Kit (TianGen, China). Gene Calow_0296 was amplified from the genomic DNA of *C. owensensis* using the primers of CoGH1A—F 5′-GCCGCGCGGCAGCATGAGTTTTCCAAAAG-3′ and CoGH1A—R 5′-GCG GCCGCAAGCGTTTATGAATTT TCCTTTAT-3′. The PCR product was then purified and separately treated with 0.5 IU of T4 DNA polymerase (Takara) and 5 mM dATP at 37 °C for 30 min. T4 DNA polymerase was then inactivated by incubating at 75 °C for 20 min, and vector pETke and the treated insert gene were annealed at 22 °C for 15 min. After that, recombinant plasmids were transferred into TOP10 competent cells, and then cultured by Lysogeny Broth (LB) agar plates with kanamycin (50 μg mL^−1^). After colony PCR and sequencing validation (Sangon, Shanghai, China), target fragments were proved to have imported into the vectors. Then positive recombinant plasmid was extracted by TIAN prep Mini Plasmid Kit (TianGen) and transformed into *E. coli* BL21 (DE3).

### Heterologous expression and purification of CoGH1A

The transformed cells were incubated overnight on LB agar plate with kanamycin at 37 °C. Single colony was picked from the plate and cultivated in liquid LB medium with kanamycin in an incubator shaker (220 rpm, 37 °C) for 12 h. The recombinant bacteria were inoculated into fresh liquid LB medium (1 L) with kanamycin and incubated in the incubator shaker (220 rpm, 37 °C) till OD600 of about 0.6–0.8. Then the isopropyl-d-thiogalactopyranoside (IPTG) with a final concentration of 0.1 mM was added to the broth for further 12-h cultivation at the conditions of 16 °C and 160 rpm. Cells were collected and resuspended in lysis buffer (50 mM Tris–HCl, pH 7.5, 300 mM NaCl), and then lysed at 4 °C by Selecta Sonopuls (JY92-IN, Ningbo Scientz Biotechnology, Ningbo, China). After centrifugation (20,000 rpm for 20 min), the supernatant was applied to a His-Tag Ni-affinity resin (National Engineering Research Centre for Biotechnology, Beijing, China) pre-equilibrated with binding buffer (50 mM Tris–HCl pH 7.5, 300 mM NaCl). The column was washed with 10 mL binding buffer to remove the nonassociative proteins. Fusion protein (CoGH1A) was eluted by elution buffer (50 mM Tris–HCl, pH 7.5, 300 mM NaCl and 150 mM imidazole). The purified CoGH1A was confirmed by sodium dodecyl sulfate-polyacrylamide gel electrophoresis (SDS-PAGE). The protein concentration was measured as described by Bradford [44] using bovine serum albumin as standard. The quaternary structure of purified CoGH1A was analyzed through gel filtration chromatography. The molecular mass (MW) was estimated by the calibration curve of log (MW) vs. elution volume.

### Assay activities of CoGH1A on different substrates

Substrate specificity of the CoGH1A was estimated by incubating the diluted enzyme in 50 mM citrate buffer (pH 6.0) containing 1 mM *p*-nitrophenol-glycosides (*p*NPGal, *p*NPGlu, *p*NPC, *p*NPX, *p*NPAr, or *p*NPM) or disaccharides (cellobiose or lactose) or 1 % (w/v) glycan (locust bean gum, synanthrin, cotton, filter paper, or carboxymethyl cellulose) at 75 °C for 5–30 min. The released *p*-nitrophenol (from *p*-nitrophenol-glycosides), glucose (from cellobiose and lactose), and reducing sugars (from glycans) were, respectively, measured by spectrophotometer (at 400 nm) [16], high-performance liquid chromatography (HPLC), and DNS method [45]. One unit of enzyme activity was defined as the amount of protein capable of releasing 1 μmol *p*NP, glucose, or reducing sugars from the substrates (releasing 2 μmol glucose from cellobiose) per minute.

### Characterization of CoGH1A

The optimum pH for CoGH1A was determined in the range of 4–8 at the temperature of 75 °C in citrate buffer. The optimum temperature for CoGH1A was determined in the range of 40–100 °C at the pH 5.5 in citrate buffer. Thermostability was investigated after incubation of the samples at different temperatures in pH 5.5 citrate buffer. Samples were withdrawn at appropriate time intervals and the residual activity was measured as described above. The effects of metal ions on CoGH1A activity were determined at 75 °C and pH 5.5. The *p*NPGal with the concentration of 1 mM was used as a substrate. The kinetic parameters on the substrates of *p*NPGal, *p*NPGlu, *p*NPC, *p*NPX, and cellobiose were analyzed at 75 °C and pH 5.5 using various concentrations of each substrate for enzyme activity assay.

### Hydrolysis of the steam-exploded corn stover

Synergetic hydrolysis by the commercial enzyme cocktail Cellic CTec2 (Novozymes) and CoGH1A was carried out in pH 5.5 acetate buffer with the initial steam-exploded corn stover concentration of 20 g L^−1^. The reaction volume was 1 mL in a 2-mL Eppendorf tube, which was sealed by winding parafilm after closing the lip, and put in a water bath at 60 °C for 72 h. The loading rates of CTec2 and CoGH1A were, respectively, 10 FPU g^−1^ biomass and 10 or 20 U_cellobiose_ g^−1^ biomass. Hydrolysis of steam-exploded corn stover using CTec2 alone in the same condition was implemented as a control. The produced sugars were quantified by high-performance liquid chromatography (HPLC) with a pump of LC-20AT (Shimadzu, Japan) and a refractive index detector (RID-10A, Shimadzu, Japan). A Hi-Plex Ca column (7.7 × 300 mm, Agilent Technology, USA) was used for the HPLC analysis. HPLC-grade water was used as the mobile phase at a flow rate of 0.6 mL min^−1^ at 85 °C.

### Lactose hydrolysis and transgalactosylation

Lactose hydrolysis and transgalactosylation by CoGH1A were carried out, respectively, at the initial lactose concentrations of 40 and 500 g L^−1^, in citrate buffer of pH 5.5 at 70 °C with the β-galactosidase dosage of 2.5 U_lactose_ mL^−1^. Quantitative analysis of the sugars was carried out by HPLC. Glucose, galactose, and lactose in the mixtures were identified by comparison of their retention times with those of the standards and quantified from the peak area calibrated against sugar standards. In this HPLC experiment, the disaccharides such as lactose, galactobiose, and allolactose could not be separated. The retention time of these disaccharides was almost the same. Therefore, the total disaccharides were quantified using the lactose as standard. The GalOS yield was calculated according to the following formula:

$$GalOS\,\text{yield}\,{(\% ) = }\,\frac{{\text{mass}\,\text{of}\,\text{initial}\,\text{lactose} - {\text{mass}}\,{\text{of}}\, ( {\text{disaccharides}}\,{ + }\,{\text{glucose}}\,{ + }\,{\text{galactose)}}}}{{{\text{mass}}\,{\text{of}}\,{\text{initial}}\,{\text{lactose}}}} \times 100\,\%.$$

This formula is based on the fact that the sum of all sugars (glucose, galactose, disaccharides, and GalOS) was approximately equal to that of initial lactose [52]. According to this formula, the GalOS yields given in this work do not include the disaccharides.

### Structure and active site prediction

The structure and active site were predicted for analyzing the difference between CoGH1A and other GHs whose structures were resolved. The amino acid sequence of CoGH1A was blasted in the Protein Data Bank (PDB) database (http://www.rcsb.org).

Multiple sequence alignment of CoGH1A with the resolved proteins was performed using the ClustalX2 software and depicted online by ESPrit 3.0 (http://www.espript.ibcp.fr/).

## Abbreviations

GH: glycoside hydrolase
FPA: filter paper activity
CMC: carboxymethylcellulose
*p*NPGal: *p*-nitrophenyl β-d-galactopyranoside
*p*NPGlu: *p*-nitrophenyl β-d-glucopyranoside
*p*NPC: *p*-nitrophenyl β-d-cellobioside
*p*NPX: *p*-nitrophenyl β-d-xylopyranoside
*p*NPM: *p*-nitrophenyl β-d-mannopyranoside
*p*NPAr: *p*-nitrophenyl α-l-arabinofuranoside
HPLC: high-performance liquid chromatography
GalOS: galactooligosaccharides
